# Vaccine suspension, risk, and precaution in a pandemic

**DOI:** 10.1093/jlb/lsab036

**Published:** 2022-03-16

**Authors:** Jonathan Pugh, Dominic Wilkinson, Ian Kerridge, Julian Savulescu

**Affiliations:** University of Oxford, The Oxford Uehiro Centre for Practical Ethics, Oxford, UK; University of Oxford, The Oxford Uehiro Centre for Practical Ethics, Oxford, UK; University of Sydney, School of Public Health and Royal North Shore Hospital, Sydney, Australia; University of Oxford, The Oxford Uehiro Centre for Practical Ethics, Oxford, UK

**Keywords:** vaccination, COVID-19, ethics, precautionary principle, paternalism, public health ethics

## Abstract

In early 2021, cases of rare adverse events were observed in individuals who had received the Astra Zeneca COVID-19 vaccine. Countries around the world differed radically in their policy responses to these observations. In this paper, we outline the ethical justification for different policy approaches for managing the emerging risks of novel vaccines in a pandemic. We begin by detailing the precautionary approach that some countries adopted, and distinguishing ethical questions regarding the management of known and unknown risks. We go on to outline the harms of adopting a highly precautionary approach in a pandemic context, and explain why an appropriate policy approach should accommodate the benefits as well as the risks of vaccination. In the final section, we outline three policy approaches that can accommodate the different benefits of vaccination, whilst taking into account the harms of precaution. Whilst we do not set out to defend one particular policy approach, we explain how different moral theories lend different degrees of support to each of these different approaches. Our analysis elucidates how fundamental value conflicts in public health ethics played out on the global stage of vaccine policy.

##  

The COVID-19 pandemic has seen the rapid development of a number of different vaccines. Although these vaccines passed the safety standards required for regulatory authorisation, our understanding of the safety profile of these novel vaccines was somewhat time-limited. As time passed, and the vaccines were rolled out to a greater number of people, we began to learn more about rare adverse events associated with the vaccines.

In March 2021, a number of countries suspended the use of the Astra Zeneca vaccine on the basis of reports of recipients experiencing thrombotic events.^1^ At the time of the initial submission of this paper in May 2021, some countries (such as Germany) had reversed initial suspensions in order to accelerate vaccine uptake;^2^ others (such as Sweden and Spain) had introduced age-limited restrictions on the use of the vaccine, whilst others (such as Denmark) continued with across the board suspensions.^3^ The UK government did not initially suspend the use of the vaccine, but later went on to suggest that those under the age of 30 should be offered an alternative to the Astra Zeneca vaccine^4^ (this age limit was later increased to 40).^5^

The management of emerging risks is part and parcel of the post-market entry for any medical therapy. Market entry is initially authorised on the basis of phase 3 trial safety and efficacy data, and then post-market safety and efficacy data is gathered in phase 4 trials. Although phase 2/3 trials have the power to detect frequent adverse effects (and therefore create an upper bound on the uncertainty of the risks of new treatments), data concerning rarer events may later arise suggesting that a new therapy should be withdrawn.

The ethical questions raised by the management of such emerging risks arose in a particularly acute form in the COVID-19 pandemic, given the significant pressure to develop novel vaccines. The phase 3 trials that formed the basis for market authorisation investigated the safety profile of the vaccines over a relatively short space of time, and only in a certain number of participants. Although such trials are more likely to miss rare adverse events that take time to manifest, there is a strong justification for approving vaccines even in the light of time-limited evidence in an emergency. In such circumstances, there is a trade-off. A novel intervention that is introduced earlier in a public health emergency may save more lives if it is effective, but there is a higher possibility that the intervention will need to be withdrawn on the basis of safety concerns missed in the phase 3 trials. Alternatively, an intervention that is introduced following more robust phase 3 trials, will have a lower probability of later withdrawal, but it will also reach the market at a later stage given the longer time it takes to run more robust trials.

The ethical question that we are aiming to address in this paper is how we ought to manage and reconcile the diverse range of risks (and uncertainty about risk) that arise in regulating novel vaccines in a global pandemic. When are doubts about the safety of the vaccine sufficient to justify the suspension of its use, or even the withdrawal of authorisation in a global pandemic? When are they sufficient to justify other risk mitigation strategies, such as employing more restrictive patient selection criteria, and issuing side-effect warnings? How does the urgency of responding to a public health emergency alter the way that we weigh and balance these risks?

In addressing these questions, we shall outline policy approaches for managing the emerging risks of vaccination in a pandemic. We shall begin by distinguishing between uncertain and known risks, with reference to observations of rare side effects following the use of the Astra Zeneca vaccine, and detailing the precautionary approach that some countries took to the management of this emerging risk. We then highlight problems with this precautionary approach, before outlining three policy approaches for managing the risks of vaccination in a pandemic that are sensitive to both the harms of precaution and the various benefits of vaccination, as well as their attendant risks. We outline how these policy approaches could accommodate a precautionary interpretation, but argue that some moral theories will support alternative interpretations.

Throughout the paper, we limit our discussion to the use of vaccines in individuals who were well represented in the initial COVID-19 vaccine phase three trials samples. Our arguments do not necessarily apply to the use of vaccines in groups (such as children) that were not represented in the trials that were invoked to justify the initial authorisation of the vaccines.

Many of the issues with which we are concerned in this paper are closely related to those that have arisen elsewhere prior to the COVID-19 pandemic. For instance, there was a great deal of bioethical scholarship about how to manage uncertainty in the development of vaccines, and the use of unproven therapies in response to the 2014 Ebola epidemic, as well as the HIV epidemic.^6^ Outside of the context of previous epidemics, trials of many other medical interventions have previously encountered unexpected adverse events, and there is a voluminous literature on risk/benefit evaluations in the research context,^7^ in addition to more targeted discussions of the ethics of stopping clinical trials early.^8^

Nonetheless, the suspension of the Astra Zeneca vaccine in the COVID-19 pandemic raises novel issues for a number of reasons. First, unlike many of the unproven interventions considered in previous epidemics, the Astra Zeneca vaccine had demonstrated sufficient safety and efficacy in the interim analysis of phase 3 trials to warrant authorisation for its use in mass vaccination programmes. Second, the significant rare adverse events were discovered following the initiation of these mass vaccination programmes. Decisions about suspension and withdrawal thus concerned the question of whether to suspend an intervention that was being widely used outside of a clinical trial, becoming a matter of national policy rather than research ethics governance alone. It is therefore important to consider whether the duties of researchers towards their study participants translate to the duties of policy-makers in regulating vaccines in a pandemic. Finally, the scale of the COVID-19 pandemic meant that many different national regulatory bodies faced decisions about suspending the use of the vaccine. As we shall detail in section I, these decisions were far from uniform, and there was a lack of transparency about how these decisions were made. For all these reasons, this series of events warrants its own focused ethical analysis.

## I. UNCERTAIN RISK AND KNOWN RISK—A CASE STUDY OF THE ASTRA ZENECA COVID-19 VACCINE

When regulators observe serious adverse events in a vaccinated population, it is important to establish whether these events were directly caused by the vaccine. When such unanticipated events first emerge, they give rise to ‘uncertain risks’ –there is some sort of association between the vaccine and an adverse event, but we do not yet fully understand it, and we do not know the degree of risk that it might connote. This raises a challenge for policy-makers; whilst it would clearly be unethical to continue with a novel intervention when there is clear and compelling evidence that its risks outweigh its benefits, the correct course of action is far less clear when the evidence of harm is inconclusive, and the potential benefit of the intervention is very large.^9^

### I. A. Uncertain Risks

Differences between the management of known risks and uncertain risks can have important implications for the moral justification of different risk management strategies. In particular, the management of uncertain risk raises at least one question that the management of known risks does not: should we continue to allow people to expose themselves to unknown risk whilst we gather more evidence, or should we suspend the use of the intervention in question until we fully understand the evidence concerning this new risk?

This became an extremely pressing question in March 2021 when, months after initially approving the Astra Zeneca vaccine, a number of European countries suspended its use following reports of individuals experiencing thrombotic events after receiving the vaccine. We provide some of the details of this series of events in Box 1.

**Box 1.**  *The Astra Zeneca Suspension—A Brief Narrative March–May 2021*

A preliminary review from The European Medical Association’s (EMA’s) in early March suggested that the reported events included cases of pulmonary emboli (including one reported fatality), and deep vein thrombosis.^10^ This initial report went on to point out that the ‘number of thromboembolic events in vaccinated people is no higher than that seen in the general population’, citing 22 cases of thromboembolic among the 3 million people vaccinated with the AstraZeneca in the European Economic Area as of the 9^th^ March. Nonetheless, a number of European countries chose to suspend the use of the vaccine on March 15^th^/16^th^. Others, such as the UK chose not to suspend the use of the vaccine.

On 16^th^ March, the German health authorities released a statement explaining that they were suspending the use of the vaccine because of seven reported cases of severe cerebral venous thrombosis (SCVT) in the vaccinated population of 1.6 m. Crucially, the report notes that this number is ‘statistically significantly higher than the number of cerebral venous thromboses that would normally occur in the unvaccinated population’.^11^ Accordingly, the German report suggested there was a stronger association between SCVT and the Astra Zeneca vaccine than the association between the vaccine and generalised thromboembolic events detailed in the early EMA report cited above.

A later report by the EMA on the 18^th^ March confirmed that the EMA found that the ‘vaccine is not associated with an increase in the overall risk of blood clots’^12^; this led some (but not all) countries to reverse their suspensions and resume their use of the vaccine.^13^ By the end of March, the EMA reported that ‘a causal link with the vaccine is not proven, but is possible and further analysis is continuing’; however, at this point, the head of the EMA told the media that there is no evidence that would support restricting the use of AstraZeneca’s coronavirus vaccine in any population.^14^ Nonetheless, some countries including Germany and France continued to suspend the use of the vaccine in certain populations. On the 7^th^ April, the EMA’s safety committee concluded that unusual blood clots with low blood platelets should be listed as very rare side effect of the Astra Zeneca vaccine, but that the benefits of the vaccine continued to outweigh its risks.^15^ This conclusion was reached in the basis of an analysis of 62 cases of cerebral venous sinus thrombosis and 24 cases of splanchnic vein thrombosis reported in the EU drug safety database by the 22^nd^ March, in a vaccinated population of roughly 25 million.

By the end of April, data began to emerge that the observed cases of SCVT with low platelets had a higher incidence in younger adults.^16^ However, the EMA maintained that the benefits of the Astra-Zeneca vaccine outweighs its risks for all age groups.^17^

As of mid-May, there is some degree of consensus regarding the pathogenesis, diagnosis, and management of thrombotic events amongst recipients of the Astra Zeneca vaccine,^18^ and a population cohort study has confirmed a higher incidence of such events in recipients of the vaccine, than in the general population.^19^

Nonetheless, different countries have adopted quite different policies with respect to the use of the Astra Zeneca vaccine between March and May 2021. Some countries, initially resumed the use of the vaccine without restrictions after the EMA judgement.^20^ On 31^st^ March, Germany restarted its roll-out, but only in high priority groups and in people aged over 60.^21^ Other countries such as Spain, Italy and Belgium also restricted the use of the vaccine to the elderly.^22^ However, in early May, German authorities abolished the age limits on the use of the vaccine in order to accelerate vaccine uptake.^23^ In stark contrast, the UK did not initially suspend the vaccine, but on the 7^th^ April it announced that those under the age of 30 would be offered an alternative vaccine.^24^ Finally, some places have ceased their rollout of the Astra Zeneca vaccine. Denmark ceased their roll-out on 14^th^ April,^25^ whilst many Canadian provinces attracted criticism in Mid-May for continuing to pause first does of the vaccine.^26^

[Fn fn24] [Fn fn25] [Fn fn26]

Some countries adopted a precautionary strategy by suspending the use of the Astra Zeneca vaccine at an early stage, whilst investigations into the reported thrombotic events were ongoing. Such a suspension allowed time for further evidence-gathering so that the magnitude of the problem could be accurately assessed, without exposing further people to the uncertain risk of harm in the meantime. In this regard, these countries adopted the spirit of the Wingspread formulation of the precautionary principle which states:

When an activity raises threats of harm to human health or the environment, precautionary measures should be taken even if some cause and effect relationships are not fully established scientifically. In this context the proponent of an activity, rather than the public, should bear the burden of proof.^27^

When there are available, safe, and effective alternative interventions, suspending a novel intervention following the observation of unexpected rare adverse events will not mean that patients are prevented from receiving treatment. Little may be lost by suspending in such circumstances. Indeed, Astra Zeneca was not the only approved vaccine available in early 2021, and other vaccines had demonstrated high degrees of efficacy in phase 3 trials.^28^ So, a decision to suspend one vaccine might have simply translated into the use of potentially safer alternatives. However, in the setting of the pandemic, with finite vaccine supplies and high demand, the suspension of the Astra Zeneca vaccine would predictably delay vaccination for some individuals (we return to this consideration below). The Astra Zeneca vaccine also had a number of well-documented practical advantages over available alternatives at this time, including ease of storage and cost.^29^ Indeed, in some countries, such as Australia, national vaccination strategy relied heavily on the use of the Astra Zeneca vaccine, until the discovery of adverse events prompted a significant shift in strategy. Media reports suggest that Australia had originally planned to manufacture 50 million doses of the Astra Zeneca vaccine. However, the government brought 20 million doses of the Pfizer vaccine in April 2021, just after announcing that Australia would stop using the AstraZeneca vaccine for people aged under 50.^30^

One argument in favour of adopting a precautionary approach to uncertainty about risks of vaccination in a pandemic is that doing so may help to ensure that these reported events do not unduly undermine confidence in the vaccine,^31^ or trust in the medical profession.^32^ Making ‘safety’ an absolute priority in the response to these reports of new adverse events might help the public to have confidence in the vaccines that are being used in the pandemic response going forward.

However, at the time, it was unclear whether the precautionary approach might alternatively undermine long-term confidence in the Astra Zeneca vaccine. Indeed, media reports at the time suggested that some patients in the UK cancelled vaccine appointments following the suspension of the vaccine.^33^ Similar concerns were later raised about the uptake of mRNA vaccines in the USA following the pause of the Johnson and Johnson vaccine.^34^ The precautionary approach may inadvertently communicate the message that the vaccine is not sufficiently safe, even if the evidence gathered during the suspension establishes that there is no causal relationship between the observed adverse events and the vaccine. Furthermore, this may impact upon vaccination rates for unrelated diseases; for instance, in Australia, there is a concern that public discourse about the safety profile of the Astra Zeneca vaccine has led to a decrease in flu vaccination uptake.^35^

Ultimately, the effect of a suspension on general vaccine confidence is an empirical issue. However, this example suggests that there is a very delicate balance to be struck in public health communication of this sort, and it is paramount that messaging here is as clear as possible. We should not simply assume without evidence that the precautionary approach is the best way to secure long-term public confidence in vaccination.

To conclude this section, uncertain risks are a particular issue when there is little available scientific data about a novel vaccine and an unforeseen adverse event. In other circumstances, uncertainty about risk can also be generated by misinformation and fraud. This has been a major issue with the novel COVID-19 vaccines in the public sphere,^36^ and is historically familiar with more established interventions such as the MMR vaccine.^37^

### I. B. Known Risks

There are further arguments in favour of a broadly precautionary approach that apply to the management of known risks as well as uncertain risks. As the narrative of the Astra Zeneca suspension suggests, the EMA eventually concluded that unusual blood clots with low blood platelets should be listed as a very rare side effect of the Astra Zeneca vaccine. When we obtain evidence that is sufficient to establish new ‘known risks’, the question becomes whether we should place further regulatory constraints on the use of the vaccine to manage these risks. Such management strategies might include the use of stringent patient selection criteria. However, at the extreme end of the spectrum, it could also include the withdrawal of the vaccine.

An advocate of a precautionary approach might claim that for both known and unknown risks it is always right to ‘put safety first’—perhaps ‘you cannot be too careful’, and we should simply not expose people to certain kinds of risks. However, there is a significant problem with this argument.

## II. THE HARMS OF PRECAUTION

A common criticism of precautionary principles is that they overlook the harms of precaution. These harms are particularly salient in a pandemic.^38^ In such circumstances, time is lives; a week spent unvaccinated is a week spent living with a higher mortality risk of COVID-19. You *can* be too careful in minimising one kind of risk when doing so involves leaving people exposed to a much greater risk.

There are four key factors that contribute to the harms of precaution in the COVID-19 context, many of which differed across countries. Together they constitute a four-factor test that can enable policymakers to quantify the harms of precaution in a pandemic context.

The first is how many people will be delayed in receiving a vaccine as a result of a vaccine suspension or withdrawal. As detailed above, there are a number of different vaccines available in some countries, so the suspension or withdrawal of one may not wholly prevent vaccination; however, at least some people’s vaccinations could be delayed. Moreover, if the suspension of the vaccination serves to undermine long-term confidence in the vaccine, then this will further diminish the number of people who will receive it.

The second is the mortality risk of the people who would be delayed in receiving a vaccine by virtue of the suspension or withdrawal of a vaccine. For example, at the time of the European suspension of the Astra Zeneca vaccine, those aged 56 and over in England were being invited to book appointments for vaccination. A recent study suggests that the average risk of death for (unvaccinated) 55–59 year-olds infected with coronavirus is roughly 0.3%.^39^ So, suspending the use of the vaccine would have delayed protection for a relatively low risk group in this situation. However, this would still translate to some preventable deaths. In the week ending 5^th^ March 2021 alone, data from the Office of National Statistics (ONS) suggests that there were 53 deaths that included COVID-19 on the death certificate in this age-band alone in England and Wales.^40^

Notably, in countries that have not yet vaccinated older age groups, the risks of a suspension will be higher. The study cited above suggests that (unvaccinated) 70–74 year olds infected with the coronavirus have an average risk of death of 1.7%—for those over 80, the risk is 8.3%. Moreover, although age is a strong predictor of COVID-19 morbidity and mortality, this risk may be heavily influenced by other factors. For instance, studies have suggested that immune suppression is a particularly strong factor in determining COVID-19 mortality risk in patients who have received haematopoietic stem cell transplantation.^41^

The risks in the above passage concern an *infected* individuals’ mortality risk. A third factor that is relevant is the prevalence of the virus at the time of vaccination delay. In times of higher prevalence, we can expect a larger number of infections and therefore deaths. According to ONS estimates at the time of the Astra Zeneca suspension in Europe, there was a low prevalence of the virus in England, around 1 in 270 (0.37%).^42^ However, in countries with a higher prevalence, suspension of the vaccine would lead to more deaths. At the time of the suspension there was a wide prevalence range in countries across Europe according to data from the European Centre for Disease Prevention and Control, with prevalence rates ranging from 15 to 1′521 per 100′000 people (0.015%—1.521%), with an average of 434 per 100′000 (0.434%).^43^

If the vaccine under consideration requires two doses, a final factor to consider is the number of people who have received one dose of the vaccine, but not a second. A withdrawal of the vaccine would potentially prevent some people from accessing a second dose from the same vaccine. Indeed, some countries that suspended the use of the Astra Zeneca vaccine (such as France) recommended that different vaccines should be used for the second dose of individuals who had received only a first dose of the Astra Zeneca vaccine.^44^ Yet, as the World Health Organisation noted, at this time there was no direct data on the interchangeability of these vaccines, although trials were under way.^45^ Moreover, even if the same vaccine might be used after a suspension, a lengthy suspension may extend the dosing period for some individuals, potentially diminishing the efficacy of the vaccine.

## III. MANAGING THE RISK AND BENEFITS OF VACCINATION IN A PANDEMIC: THREE POTENTIAL POLICY APPROACHES

Governments have a responsibility to protect their citizens from harm, and this includes the harms of precaution. However, if it is clear that a new medical technology poses a greater risk of harm than benefit, then there are strong moral reasons for governments (or authorised regulatory bodies) to withdraw the use of that intervention. These reasons can be paternalistic; that is, the justification of withdrawing a risky vaccine might appeal to the moral reasons to prevent people from making harmful choices. However, other motivations are possible. A further rationale in support of withdrawal might appeal to consideration of justice; ineffective and risky procedures may require the use of medical resources that could be put to far better use.

As the risk/benefit balance of the intervention becomes less clear, so too does the appropriate policy response. Indeed, in the circumstances surrounding the Astra Zeneca vaccine there are a number of reasons why it is problematic to claim that there is a clear, objective quantitative threshold that can or should determine policy responses. First, the direct and indirect benefits of vaccination and the disvalue attached to the potential adverse event are unlikely to be uniform across individuals. Second, each individual’s degree of risk will be influenced by a wide range of factors (as detailed in the previous section) that will likely not be captured by a population level risk assessment of the vaccine in different groups. Third, there are also a number of obstacles to reliably estimating an individual’s morbidity/mortality risk from a rare adverse event following vaccination. There are thus considerable conceptual and practical challenges for policy-makers responding to observations of rare events following novel vaccinations in a pandemic. However, these challenges do not obviate the need to make a policy response.

As detailed in section I, a popular policy response in the case of the Astra Zeneca vaccine was to suspend the use of the vaccine as soon as there was evidence that it could be causing a serious adverse event. This approach can be supported by different sorts of precautionary principles. The most simple precautionary principle in this regard might be the following:

*Simple Precaution*: The avoidance of vaccine-associated harms should take lexical priority over the benefits that might be achieved through the continued use of the vaccine.

The simple precautionary approach is broadly analogous to what Kimmelman describes as ‘maximin’ study designs in the research context; such studies aim to minimise harms from the worst possible outcome from the trial intervention.^46^

Although such a simple precautionary principle lends support to the decision to suspend the Astra Zeneca vaccine, this principle is misguided. It is crucial that a policy response to adverse events following vaccination is sensitive to the harms of precaution detailed above. As we shall explain below, the decision to suspend the vaccine can be supported by far more plausible precautionary principles that at least take into account all of the morally relevant features of this decision. Indeed, when discussing the broadly analogous maximin strategy in the research context, Kimmelman explicitly points out that such a strategy would be inappropriate in the context of a public health crisis, in view of the catastrophic losses that can be expected in the absence of treatment.^47^

In the remainder of this section, we shall outline three potential approaches that are sensitive to these factors, and that policy-makers might adopt in forming a decision about whether to continue using a vaccine following the observation of adverse events. To begin this discussion, it is important to first be clear about the relevant comparisons to make when determining the risk/benefit balance of a vaccine in a pandemic.

### III. A. Acceptable Risk Thresholds, Risk of Harm Comparisons and Risk/Benefit Assessments

One common approach evident in the public debate about the emerging risks of the Astra Zeneca vaccine assessed whether these risks fell above or below some threshold of ‘acceptable risk’ that an intervention or technology may not exceed. For example, in a quite different context, the Health and Safety Executive adopted such an approach in determining levels of broadly acceptable and maximum tolerable individual risks of fatality for nuclear plants.^48^

A major challenge facing this simple acceptable risk threshold approach is how to choose a non-arbitrary threshold of acceptable risk. One solution to this issue is to base the thresholds we employ on the kinds of risk that are deemed to be acceptable in other contexts. Indeed, this is the approach adopted by the minimal risk threshold that is often invoked in the research context, according to which a trial poses only minimal risk if the probability and magnitude of harm anticipated in the trial are not greater than those ordinarily encountered in daily life.^49^ For example, in the medical context, one relevant comparator might be the fatality risk of general anaesthetic (roughly 1 in 100′000), or the risks associated with over the counter medicines (such as oral contraceptives, which are also associated with fatal thrombotic events, with one study suggesting incidences ranging from 5.1–9.1 cases per million women per year).^50^

As Kimmelman has pointed out, one problem with appealing to acceptable risk thresholds is that paradigmatic examples of acceptable risk unavoidably embody cultural preferences and the social influences upon those involved in setting the threshold.^51^ A further problem with invoking this standard in a pandemic is that the risks that might be acceptable in the extraordinary circumstances of a pandemic might be quite different to those that are acceptable outside of that context. The reason for this is that in a pandemic, the status quo is such that we each face some baseline level of risk of mortality and morbidity from the pandemic disease, in the absence of an effective preventative intervention.

In view of this, perhaps the most plausible ‘acceptable risk’ threshold to consider in this context is an individual’s risk of morbidity/mortality from COVID-19. Indeed, some of the discussion in the public sphere adopted this sort of acceptable risk approach. For example, a BBC infographic compared the risk of serious harm due to a side-effect from the Astra Zeneca vaccine for a 25-year old (estimated to be 11 in a million) and a 55-year old (estimated to be 4 in a million), and the risk of dying with coronavirus for these individuals (23 in a million and 800 in a million respectively, according to the infographic).^52^

Such comparisons provide us with what we might call a risk of harm comparison score:

*Risk of Harm Comparison Score*

Overall risk of mortality or morbidity in pandemic without vaccine - Risk of morbidity or mortality from vaccine side-effect = **Risk of Harm Comparison Score**

As noted in section II, an individual’s overall risk of morbidity or mortality from a pandemic disease should be understood to be a function of both their risk of morbidity or mortality from the disease itself, and their probability of becoming infected in the first place. The latter variable will fluctuate in different stages of the pandemic, as the prevalence of the virus changes over time. This score could be calculated for quite different populations. Of course, the less information we have about the relevant risks, the wider the confidence interval around the estimated risk of harm comparison score for a particular group.

When an individual’s risk from vaccination is greater than their risk from COVID, then the risk of harm comparison score can be sufficient for establishing that the vaccine can be expected to do more harm than good. However, the problem with this score is that it is incomplete; it only compares the potential harms of vaccination for the individual against the harms of not being vaccinated. It fails to accommodate the extent of the wider *benefits* of vaccination.

Effective vaccines can provide a *direct benefit* to the recipient herself, insofar as they protect the recipient from morbidity and mortality associated with the pandemic disease. However, in addition, vaccination often has the *indirect benefit* of preventing transmission of the targeted infectious pathogen to others. The indirect benefits of vaccination are not wholly altruistic, since some indirect benefits of vaccination also accrue to vaccinated individuals. For instance, a vaccinated individual may indirectly benefit from the fact that the vaccine has lowered their risk of transmitting the virus to vulnerable individuals with whom they share a close relationship. Alternatively, in some countries, certain societal freedoms may only be extended to those who can prove that they pose a sufficiently low risk of transmission. At the time of writing, we do not yet have robust data about the extent to which the approved COVID-19 vaccines prevent transmission of the virus. However, there is some promising data in this regard.^53^

To begin, let us consider only the direct benefits of vaccination. When an individual’s risk from vaccination is lower than their risk of dying from COVID, the direct benefit of vaccination *may* outweigh its risks, but this comparison alone is not sufficient to establish that it will do so. In order for the benefits of vaccination to outweigh its risks in these circumstances, the vaccine must also be sufficiently *effective* in reducing an individual’s risk of dying from COVID. Only then will the taking the vaccine be worth the risk for that individual.

Accordingly, a risk/benefit assessment of when a vaccine is in an individual’s best interests must incorporate further factors, that allow us to estimate how much good the vaccine can be expected to do in some group:

*Direct Benefit Formula:*

Expected benefit from vaccine – expected harm = **direct benefit score**

According to this formula, the further above zero the direct benefit score of a vaccine, the greater the extent to which its expected benefits will outweigh its expected harms. The further below zero the direct benefit score of a vaccine, the greater the extent to which its expected harms will outweigh its expected benefits.

The expected benefit and expected harms of vaccination are a function of both the degree of the effect in question, and its probability. The direct benefit score differs from the risk of harm comparison score because the expected benefit from vaccination depends not only on (i) the individual’s risk of morbidity/mortality without vaccination but also (ii) the effectiveness of the vaccine in reducing that risk. In the specific context of COVID-19, there may not be huge differences between an individual’s risk of harm comparison score and their direct benefit score, as the COVID-19 vaccines are highly effective in preventing severe illness and death. Notably though, some of the variables that affect morbidity/mortality risk (such as age and the certain co-morbidities) may also affect the effectiveness of vaccines in different groups. An accurate assessment of the direct benefit of a vaccine for a group must be based on that group’s particular morbidity/mortality risk, and the likely effect of the vaccine in that group.

To illustrate the difference between the two scores with a completely hypothetical example, suppose an individual had a 40% risk of dying from a pandemic disease, and an available vaccine posed a 30% risk of a fatal side-effect. Such an individual’s risk of dying from the pandemic disease outweighs their risk from vaccination on the risk of harm comparison outlined at the beginning of this section. The risk of harm assessment score would be positive. However, if the vaccine is less than 75% effective, the direct benefit score will be negative for that individual; the risks associated with vaccination will not be worth taking for this individual. When this is the case, the risk of vaccination will be greater than the reduction in the individual’s disease risk that the vaccine will evince.

There are a number of complexities in calculating the direct benefit score in different groups. First, there are challenges in modelling a given individual’s risk of mortality at a given point in the pandemic, as outlined in the previous section. Furthermore, the adverse events discussed above are rare, and the risks of the virus evolve rapidly. Since it accommodates vaccine effectiveness, the direct benefit formula adds a further dimension of complexity to the simple risk of harm comparison score. Whilst initial studies have established the efficacy of approved vaccines across different age groups, these studies were not sufficiently powered to provide a fine-grained sub-analyses of the efficacy of vaccines in particular demographic groups. Yet, despite all of the various complexities outlined in this paragraph, some countries did decide to suspend the Astra Zeneca vaccine in an age-specific manner, as detailed in box 1.

Some of the risk infographics published in the public sphere accommodated the considerations that are captured by the direct benefit formula. For instance, the Winton group published three models of how to weigh the potential benefits and harms of the Astra Zeneca vaccine in periods of low, medium and high exposure risk. First, taking data from the available MHRA reports of blood clot reactions, the infographs included an estimate of the potential harms of vaccination in different age groups. For example, the model estimated that the risk of vaccination in the 60–69 age band was 0.2 cases of clotting per 100′000 people, whilst in the 20–29 age band, the risk was estimated to be 1.1 cases of clotting per 100′000.

The infographic also included data about the direct benefits of vaccination for each age group based on the number of ICU admissions that the vaccine could be predicted to prevent every 16 weeks, and assuming a fixed vaccine efficacy of 80% across all age groups. These data can be easily compared to this information about the risk of vaccination. The data about the benefits of vaccination depend a great deal on the degree of virus exposure used in each model. For instance, on the high exposure model, the model suggests that the vaccine could be expected to prevent 127.7 ICU admissions per 100′000 people in the 60–69 age band, and 6.9 per 100′000 in the 20–29 age band. In contrast, on the low exposure model, this fell to 14.1 ICU admissions in the 60–69 age band, and 0.8 in the 20–29 age band. Notice that that on the low exposure model, the vaccine is expected to cause a higher number of blood clots per 100′000 people (i.e. 1.1 cases) than ICU admissions it will prevent in this younger group (0.8).

Accordingly, the Winton Group modelling thus suggests that the Astra Zeneca vaccine may have a negative direct benefit score in younger age groups in periods of low exposure to the virus. We shall now explain how the direct benefit score could be used as a basis for vaccine policy.

### III. B. Policy 1—A Direct Benefit Score Threshold

One approach would be to use only a direct benefit score threshold to determine whether or not a vaccine should be used in a particular group. On this approach, reaching the stipulated direct benefit score threshold would be both necessary and sufficient for the permissibility of continuing to deploy a vaccine. Further precaution could be built into this policy by claiming that the lowest bound of the confidence interval surrounding the vaccine’s estimated direct benefit score must surpass this stipulated threshold.

Of course, it would also be technically possible to assess direct benefit scores on an individual basis. Individually assessed scores could be more accurate, as individuals will have access to unobserved information that allows more precise calculation of their likely benefit from vaccination. However, such a personalised approach would be highly resource intensive, and unlikely to be a practically feasible policy approach in a pandemic. Nonetheless, the possibility does raise a problem with highly precautious policy approaches using group-based risk assessments; individuals may in some cases be able to make more precise assessments of the likely benefit of vaccination in their own specific case than is possible with a group-level assessment. We return to this point in our discussion of libertarian arguments below.

The direct benefit score can be used to develop a precautionary approach that goes beyond the simple precautionary principle outlined in previous sections. Such a precautionary approach would invoke a high direct benefit score threshold. On this view, we should only use vaccinations when the benefits of vaccination to the individual outweigh its risks by a large magnitude. Accordingly, this approach might preclude the use of some vaccines that we could expect to do more good than harm. This will be so, when the expected benefits of vaccination outweigh the risks, but not to a sufficient degree to pass a high direct benefit threshold. On this approach, the avoidance of vaccine associated harms does not take lexical priority over the benefits of vaccination (as per the ‘simple precaution’ policy we rejected at the beginning of this section); however, the avoidance of such harms does take some weighted priority.

A high direct benefit threshold might also be supported by broader consequentialist considerations. Indeed, it might be argued that we should not approve a vaccine with a sufficiently high mortality/morbidity risk, even if we had a high degree of certainty that the expected benefit of the vaccine outweighed the risks for the group in question. To illustrate again with a completely hypothetical example, a 90% effective vaccine with a 50% risk of vaccine-related morbidity/mortality would still have a positive direct benefit score for individuals who otherwise face a risk of death from the disease in question greater than 55%. Yet, the use of a vaccine that caused mortality or morbidity in half of the people who received it would plausibly be damaging to vaccine confidence in the wider public, even if it was in the interests of some very high-risk individuals. Accordingly, it might be argued that we should employ a threshold direct benefit score that is higher than 0 to prevent the use of such risky but beneficial vaccines. The threshold could be set at a level that would rule out vaccines that pose a particularly high degree of risk; the direct benefit threshold could accommodate the view that we should not allow the use of vaccines that pose unacceptable degrees of risk, even if they are in an individual’s all things considered best interests.

Approaches that adopt higher direct benefit thresholds than 0 face the same issues of arbitrariness facing the acceptable risk threshold approach considered above. One non-arbitrary basis for drawing a threshold would be to appeal to empirical data about how best to protect public confidence in vaccination, and the degree of risk that people are willing to accept. Are individuals simply willing to use a vaccine that has a positive direct benefit, or are they only willing to vaccinate once the direct benefit score passes a higher threshold?

Of course, one need not adopt this sort of precautionary approach to direct benefit thresholds. Another obvious candidate for a direct benefit threshold for vaccine policy is a score of 0. This threshold would not be arbitrary in the same way as the acceptable risk thresholds considered in the previous section; vaccines that fall below this threshold pose a greater risk of harm than benefit. There is thus no individual beneficence-based justification for providing a vaccine that falls below this threshold to the group concerned. Indeed, it might be argued that permitting the use of a vaccine in individuals for whom the vaccine has a direct benefit score below 0 would amount to a violation of a duty of non-maleficence that governments might owe to their citizens in medical policy-making.

However, using only a positive direct benefit threshold to determine vaccine policy faces another deeper problem; it is paternalistic.^54^ Such thresholds prevent people from autonomously choosing to take a risk that may be beneficial to others.

### III. C. Policy 2—A Total Benefit Score Threshold

Even if the risks of a vaccine outweigh its direct benefits for individuals in a given group, the vaccination of that group may have considerable indirect benefits if the vaccine is effective in preventing viral transmission. This is particularly so if members of that group would otherwise pose a high risk of transmission. In this way, vaccination can pose a classic health problem; a population that is exposed to risk by some policy may be quite different to the population that is expected to benefit most from that policy. Indeed, in the context of the Astra Zeneca vaccine, data suggests that the direct benefit of vaccination for the elderly will be far greater than the young; not only do the elderly have a higher morbidity/mortality risk from COVID-19 than the young, they also have a lower higher risk of a thrombotic event following this vaccine than younger people. Yet, vaccinating the young may serve to provide further indirect protection to the elderly.

An alternative to using the direct benefit threshold is to base policy on the *total* benefits of the vaccine, incorporating both the direct and indirect benefits of vaccination. One way to assess the total benefit of a given vaccine is to assess its likely direct benefits and to supplement this with a model of how many deaths and cases of severe illness the vaccine can be expected to prevent in a given population. For instance, analysis from Public Health England in May 2021 suggested that the COVID vaccines had saved 11,700 lives and stopped 33,000 people becoming seriously ill in England to that point.^55^ Similarly, one might only approve the use of a vaccine that is expected to prevent a sufficient degree of morbidity and mortality in the population, whether that is through providing direct or indirect benefits. When only one vaccine is available, the relevant comparator for the vaccine’s total benefit score is the baseline level of morbidity and mortality we might expect in the absence of vaccination. However, when multiple vaccines are available, the relevant comparator for a vaccine’s total benefit score will be the total benefit score for alternative vaccines. In both cases, a total benefit approach would stipulate a certain threshold total benefit that a vaccine would have to achieve relative to the relevant comparator.

One notable feature of the total benefit approach is that a vaccine with a negative direct benefit in some groups could still potentially have a positive total benefit score. To illustrate, the direct benefit for one group could fall below 0; however, suppose that this group had a significant role in transmitting the virus. The indirect benefit of vaccinating this group could be very high, perhaps if the group in question posed a high risk of transmission, and the vaccine was very effective at reducing transmission in this group. In some circumstances then, if we could expect to prevent a significant amount of morbidity and mortality in vulnerable individuals by vaccinating another group that is not itself directly benefited by the vaccination, this may be supported by a total benefit approach. In other words, a total benefit approach might support permitting the use of a vaccine in a group even if it is likely to do more harm than good for the individuals in that group.

Ultimately, the question of whether such an approach could be ethically justified boils down to the fundamental issue of whether it can be ethical to allow individual to expose themselves to risk in order to secure benefits for others. Most obviously this can be supported by broadly utilitarian considerations. A crude utilitarian approach might support the use of a total benefit threshold in all cases, even when the risks that some individuals might be exposed to are very large.^56^ Indeed, as Malm and Navin have pointed out, mass vaccination programmes have often been defended by appealing to the aggregate well-being they promote;^57^ the standard construal of risk benefit trade-offs in medical research appears to take a similarly aggregative approach.^58^ Yet, such crude utilitarian approaches are unlikely to win widespread support; indeed, such justifications have previously been criticised on the basis that they would entail using certain individuals as mere means to benefit others.^59^

However, broadly deontological considerations can also be invoked to support permitting some individuals to expose themselves to risk in order to benefit others. This may particularly be so when the individuals in question have certain duties towards those who will benefit from their exposure to risk. For instance, certain professional roles may connote such duties; those in the medical profession might plausibly be said to have a duty to those in their care to take on certain risks if that is necessary for the protection of those in their care. More broadly, it might be argued that we all have some minimal duties of beneficence to our fellow citizens.

Yet, the deontological justification of allowing individuals to expose themselves to risk in order to benefit others is unlikely to support a simple total benefit threshold. The reason for this is that our duties of beneficence are typically understood to be limited in significant ways; a duty of beneficence does not entail that one has a duty to take on a very great risk in order to secure a marginal benefit for others. For example, the duty of easy rescue that we might have to our fellow citizens may only require that we expose ourselves to very low levels of risk to benefit others.

Accordingly, those who reject the simple utilitarian approach, and those who believe that duties of beneficence are subject to limits are likely to reject the total benefit approach. However, there is one final view that might be invoked to support it. It might be argued in a broadly libertarian vein that people should be free to make their own autonomous decisions about whether to take on certain risks, regardless of whether they have a duty to do so. Indeed, it may be unduly paternalistic to prevent people from making choices simply because those choices expose them to risks of harm.

Of course, a simple libertarian view might claim that people should be free to choose to use *any* vaccine, even if it does not promise to have an overall total benefit. As we mentioned above, there are non-paternalistic reasons of justice to prevent people from using a vaccine that poses a large risk of harm. However, if the vaccine in question is effective in achieving an overall public health benefit, these non-paternalistic reasons for withdrawal are significantly diminished. Indeed, an individual can quite rationally choose to undergo a vaccination that has a negative direct benefit score in their particular demographic group. For instance, the individual may be motivated by altruistic reasons; alternatively, their individual circumstances might give them some prudential reasons to receive a vaccine that will lower their transmission risk, perhaps because they have close and valuable relationship with a vulnerable individual. Alternatively, they may live in a society in which certain benefits are afforded to those who have been vaccinated. Finally, even if the direct benefit score for their demographic group falls below zero, an individual may be able to make a more accurate personalised assessment of the benefits of vaccination in their own individual case, due to their knowledge of personalised information about their own individual risk factors. In some cases, the individual’s direct (and indeed total) benefit score may be considerably higher than it is for the overall demographic groups to which they belong.

This suggests that a tempered version of the libertarian view may be more plausible; people should be free to choose to use any vaccine that secures an acceptable level of total benefit. There are two central premises at the heart of the tempered libertarian view. The first is that considerations of individual autonomy should trump considerations of beneficence and non-maleficence. The second is that people are able to make voluntary and informed choices in the face of uncertain information about risk. Some may be willing to accept both of these premises; if so, they may find the use of a total benefit threshold an acceptable approach to vaccine policy. But we suspect that many will find at least one of these premises objectionable; indeed, the first premise is rejected by some in the research ethics literature, who claim that there should be upper limits to the risks that healthy volunteers can consent to in biomedical research.^60^ Similarly in the context of pandemic policy, it might be argued that some (hard) paternalism is warranted in order to not only protect societal trust in the medical profession, but also to protect people from making poor choices. Such choices may include what Jansen and Wall have described as reckless (as opposed to virtuous) imprudence, which involves choosing to expose oneself to a grave risk of harm in order to accrue small benefits to others.^61^ Furthermore, it might be claimed that the medical professional’s primary duty is that of non-maleficence, and that they can never justifiably expose even a consenting individual to risk of net harm to themselves in order to benefit others. Alternatively, it might be argued that such (soft) paternalism is justified due to the significant obstacles to ensuring truly autonomous decision-making in the face of complex information about uncertain risks.^62^

These are valid concerns that we do not have space to address. We shall conclude by outlining a final hybrid policy that seeks to accommodate both the direct and indirect benefits of vaccination. However, it does so in a way that avoids the connotations of simple utilitarianism, and that need not rely on this tempered libertarian approach.

### III. D. Policy 3—A Two-Step Hybrid Approach

As detailed above, the problem with the direct benefit policy is that it precludes the possibility that individuals may have reasons to use a vaccine that is not expected to directly benefit them. Conversely, a problem with the total benefit approach is that it could justify allowing individuals to expose themselves to a large degree of risk to benefit others.

A two-step approach could be employed to prevent these issues. The first part of the two-step policy would employ a direct benefit *sufficiency* threshold. If a vaccine has a direct benefit score above this threshold, then it could permissibly be deployed.

**Part A.**

Does the vaccine pass a direct benefit sufficiency threshold?

If the answer to this question is yes, then the vaccine may be permissibly deployed. If the answer is no, then policy-makers should proceed to part B. To illustrate the first step of the hybrid policy in the context of the Astra Zeneca vaccine, given the significant COVID-19 mortality/morbidity risk in the elderly, and their low risk of serious adverse events in this age group, it may have been the case that the vaccine would have had a large enough direct benefit score in the elderly to pass even a high direct benefit sufficiency threshold. Conversely, the direct benefit score in younger age groups may not have been high enough to pass a high sufficiency threshold, given their lower COVID-19 mortality/morbidity risk, and their relatively higher risk of serious adverse events.

Nonetheless, policy three differs from policy one because passing the threshold in Part A is only *sufficient* for the permissibility of deploying a vaccine; it is not *necessary*. Accordingly, unlike policy 1, policy 3 does not preclude the possibility of allowing people to access a vaccine that does not provide a degree of direct benefit that is alone sufficient to justify permitting the use of the vaccine in that group.

One of the problems with policy 2 is that it permitted people to expose themselves to potentially large degrees of risk, in order to secure benefits for others. We noted that this may be supported by utilitarian and broadly libertarian approaches. However, one way to avoid this connotation of the total benefit approach, whilst allowing indirect benefits of vaccination to inform policy, is to adopt a two-step approach. On this approach, some lower direct benefit score is deemed necessary (but not sufficient) for the permissibility of deploying a vaccine. A vaccine that does not pass the direct benefit sufficiency threshold of Part A could yet be permissibly deployed if it satisfies *both* conditions of Part B:

**Part B.**

Does the vaccine:

(i) Pass a minimum direct benefit threshold

And

(ii) Pass a minimum total benefit threshold?

If the answer to both B(i) and (ii) is yes, then the vaccine may permissibly be deployed. On this approach, no amount of indirect benefit will be able to justify the use of a vaccine that poses a certain level of risk to the recipient herself.

With respect to (i), those who are sceptical that individuals have a duty to expose themselves to *any* risk of harm in order to benefit others, and/or those who believe the duty of non-maleficence is the most salient consideration for vaccine policy, might claim that policy-makers should employ a direct benefit threshold of at least 0 here. Such an approach would prevent the ‘mere means’ objection to the consequentialist interpretation of policy 2. This interpretation would also echo elements of Jansen and Wall’s hard paternalist fair risk/benefit restriction on clinical research, according to which ‘trials should not impose risks of harm on subjects that are not proportionate to the prospective benefits the trial presents to the subjects themselves’. As the authors point out, this (defeasible) restriction speaks against trial that are not in the best interests of the participants themselves; however, they note that the interpretation of proportionality in their restriction is sensitive to the paternalist reasons that apply in a given trial.^63^ Notably, it might be claimed that there are also reasons of justice to invoke a threshold of at least 0 in groups that have been disproportionately affected by the pandemic or subject to historical exploitation. Regardless of the justification, an approach that employs a minimum direct benefit threshold of at least 0 will serve to ensure that this two-step approach would only permit the use of vaccines that would directly benefit recipients themselves; it would prevent individuals from being exposed to high risk in order to benefit others.

If a below-zero threshold for (i) is used, then policy 3 would support the use of vaccines that pose a greater risk of harm than benefit for some groups. The further below 0 the threshold for (i) is set, the greater the risk that individuals may be exposed to in order to indirectly benefit others on this policy. Thresholds that fall far below zero may be supported by either broadly consequentialist moral reasoning, deontological theories that claim that individuals have strong duties to take even high risks for (proportionate) benefits to others, and broadly libertarian considerations. However, deontological theories may also be invoked to set limits on how far below 0 it may be permissible to set the direct benefit threshold in B, or on how great a risk it is acceptable to allow individuals to expose themselves in order to benefit others, in the name of what Jansen and Wall call ‘justified unfairness’ in the research context.^64^

It is also possible to appeal to acceptable risk thresholds that we employ elsewhere to determine the relevant sub-zero direct benefit threshold on this approach; in other domains, how much risk do we allow competent individuals to expose themselves to in order to benefit others? For instance, in discussing upper limits to risks in clinical research, London suggests that the risks of research should not exceed those involved in a risky but socially valuable profession like fire-fighting.^65^ However, this strategy faces the same issue we identified in the context of acceptable risk thresholds above; why suppose that the acceptable risk thresholds we endorse in normal life should simply transfer to the emergency situation of a global pandemic?

The higher above 0 one sets the threshold for (ii), the greater the public health benefit a vaccine that fails to pass the direct benefit sufficiency threshold in Part A will have to evince if it is to be permitted. Of course, policy 3 could be used to develop a number of (i) and (ii) threshold pairings. For instance, if an intervention were to pass a very high (ii) threshold, then it might be plausible to also adopt a lower threshold of direct benefit in (i). We might adopt a correspondingly higher threshold of direct benefit in (i) for interventions that promise smaller total benefits. Generally though, maximising consequentialist theories are more likely to support lower total benefit thresholds on this approach; on these views, we should permit interventions as long as their benefits outweigh the harms, all things considered. However, in so far as deontological theories claim that individuals only have duties to take on risks for benefits to others that are proportionate to this risk, such theories are likely to support higher total benefit thresholds.

To illustrate how the hybrid approach might have been applied to the Astra Zeneca vaccine, a precautious interpretation of this approach could set sufficiently high thresholds in both Part A and B to preclude the use of the vaccine in *any* group. A moderately precautious interpretation would set a low enough threshold in Part A to permit the continued use of the vaccine in groups that were most vulnerable to COVID-19 (such as the elderly), but sufficiently high thresholds in part B to ensure that groups would be unable to access the vaccine if its overall benefits were less certain. This approach therefore might have supported initial suspension of the vaccine only in younger age groups.

All the policies we have considered here take into account the harms of precaution. So too does the simple risk of harm comparison score that we illustrated at the outset of this section. However, the policies we have considered here go beyond the risk of harm comparison by considering the different benefits of vaccination. Policy 1 takes only the direct benefits of vaccination into account, whilst policies 2 and 3 accommodate both the direct and indirect benefits of vaccination. Unlike policy 2, policy 3 accommodates both of these benefits in a manner that does not rely on utilitarian or libertarian premises. Accordingly, we believe that policy 3 is likely to garner the most pluralistic support of the policies presented here, but different moral theories may be invoked to support higher or lower thresholds for both (i) and (ii) on this policy approach.

## IV. CONCLUSION

The association between the Astra Zeneca vaccine and rare serious adverse events has raised stark moral challenges for policy-makers. Countries have reached quite different policy positions in the light of this evidence, but the rationales for these polices have not been wholly transparent. Our analysis in this paper provides an ethical framework for understanding the justification for different policies that may be adopted in the light of emerging cases of serious adverse events following vaccination.

As noted above, the EMA maintained after its investigation of the matter that the benefits of the Astra-Zeneca vaccine outweigh its risks for all age groups, and there is emerging data to suggest that the vaccine may have considerable indirect benefits. If that is the case, then we can draw the following conclusions from our analysis. First, none of the policies outlined in the previous section support a continued complete suspension or withdrawal of the vaccine in mid 2021 if alternative vaccine supplies remain scarce. The appropriate limits on the continued use of the vaccine depend on which policy approach we adopt. Strikingly, the continued use of the vaccine in all age groups could theoretically be justified according to the three policy approaches we have outlined here. This is most obviously true of policy 2, according to which the indirect benefits of vaccination should significantly shape vaccine policy decisions. Yet, the continued use of the vaccine in all age groups can also be justified by policies 1 and 3, provided the direct benefits thresholds employed do not rise too far above zero. However, the higher the direct benefit thresholds are raised in policies 1 and 3, the more that these policies will prescribe limiting the use of the vaccine to increasingly older people.

The decision about which of these policies we should adopt, and the thresholds that we employ in implementing those policies, essentially boils down to an ethical judgement about how to deal with fundamental and inevitable value conflicts in public health ethics. Should we favour policies that best secure benefits to the community, and privilege considerations of individual autonomy, or should we prioritise non-maleficence? Our aim here has not been to settle these conflicts, but rather to elucidate quite how they have played out on the global stage, and to increase transparency about how these values might be weighed in the crucial decisions being made about how to regulate novel vaccines in a pandemic.

## Funding

This research was funded in whole, or in part, by the Wellcome Trust [Grant number WT203132/Z/16/Z], and by UKRI/AHRC Ethics Accelerator: Grant Ref: AH/V013947/1 and the Oxford Martin School. For the purpose of open access, the author has applied a CC BY public copyright licence to any Author Accepted Manuscript version arising from this submission. JS, through his involvement with the Murdoch Children’s Research Institute, received funding through from the Victorian State Government through the Operational Infrastructure Support (OIS) Program.

## Conflict of Interest Statement

JP, DW and JS are employed by the University of Oxford. is a Partner Investigator on an Australian Research Council Linkage award (LP190100841, Oct 2020–2023) which involves industry partnership from Illumina. He does not personally receive any funds from Illumina. He is also a Bioethics Committee consultant for Bayer.

## Endnotes

1 Staff, *Factbox: Countries Suspending AstraZeneca’s Vaccine after Blood Clot Reports*, Reuters, https://www.reuters.com/article/us-health-coronavirus-astrazeneca-vaccin-idUSKBN2B72J5 (accessed Mar. 15, 2021).

2
*Britain, Germany in Role Reversal on AstraZeneca Vaccine Risks*, Reuters, https://www.reuters.com/world/europe/third-wave-pandemic-appears-be-broken-german-health-minister-2021-05-07/ (accessed May 7, 2021).

3
*AstraZeneca Vaccine: Denmark Stops Rollout Completely* (BBC News), sec. Europe. https://www.bbc.com/news/world-europe-56744474 (accessed Apr. 14, 2021).

4
*COVID: Under-30s Offered Alternative to Oxford-AstraZeneca Jab* (BBC News), https://www.bbc.co.uk/news/health-56665517 (accessed May 18, 2021).

5 Engl, *Public Health. JCVI Advises on COVID-19 Vaccine for People Aged under 40* (GOV.UK), https://www.gov.uk/government/news/jcvi-advises-on-covid-19-vaccine-for-people-aged-under-40 (accessed May 19, 2021).

6 Philippe Calain, *The Ebola Clinical Trials: A Precedent for Research Ethics in Disasters*, 44 J. Med. Ethics 3–8 (2018), https://doi.org/10.1136/medethics-2016-103474; Seema K. Shah, David Wendler, & Marion Danis, *Examining the Ethics of Clinical Use of Unproven Interventions Outside of Clinical Trials During the Ebola Epidemic*, 15 Am. J. Bioethics 11–16 (Apr. 3, 2015), https://doi.org/10.1080/15265161.2015.1010996; Annette Rid & Ezekiel J. *Emanuel, Ethical Considerations of Experimental Interventions in the Ebola Outbreak*, 384 The Lancet (British Edition), 1896–99 (2014), https://doi.org/10.1016/S0140-6736(14)61315-5; *infra* note 38; Morenike Folayan, Aminu Yakubu, & Bridget Haire, et al., *Ebola Vaccine Development Plan: Ethics, Concerns and Proposed Measures*, 17 BMC Med. Ethics 1–8 (2016), https://doi.org/10.1186/s12910-016-0094-4.

7 Annette Rid & David Wendler, *A Framework for Risk-Benefit Evaluations in Biomedical Research*, 21 Kennedy Inst. Ethics J. 141–79 (2011), https://doi.org/10.1353/ken.2011.0007; Charles Weijer & Paul B. Miller, *When Are Research Risks Reasonable in Relation to Anticipated Benefits?*, 570–73 (June 1, 2004); Franklin G. Miller & Howard Brody, *Clinical Equipoise and the Incoherence of Research Ethics*, 151–65 (Apr. 2007); Alex Rajczi, *Making Risk-Benefit Assessments of Medical Research Protocols*, 32 J. Law, Med. & Ethics 338–48 (2004), https://doi.org/10.1111/j.1748-720X.2004.tb00480.x; Annette Rid, Ezekiel J. Emanuel, & David Wendler, *Evaluating the Risks of Clinical Research*, 304 JAMA 1472–79 (Oct. 6, 2010), https://doi.org/10.1001/jama.2010.1414.

8 Erik Malmqvist, Niklas Juth, Niels Lynöe, & Gert Helgesson, *Early Stopping of Clinical Trials: Charting the Ethical Terrain*, 21 Kennedy Inst. Ethics J., 51–78 (Mar. 2011), https://doi.org/10.1353/ken.2011.0002.

9 Malmqvist et al. make a similar observation in their discussion of the early stopping of clinical trials for safety reasons.

10 Ana Catarina Pinho, *COVID-19 Vaccine AstraZeneca: PRAC Preliminary View Suggests No Specific Issue with Batch Used in Austria* (Text. European Medicines Agency) https://www.ema.europa.eu/en/news/covid-19-vaccine-astrazeneca-prac-preliminary-view-suggests-no-specific-issue-batch-used-austria (Mar. 10, 2021).

11 Paul-Ehrlich-Institut – News – The Paul-Ehrlich-Institut informs, *FAQs — Temporary Suspension of Vaccination with COVID-19 Vaccine AstraZeneca* (accessed Mar. 18, 2021), https://www.pei.de/EN/newsroom/hp-news/2021/210315-pei-informs-temporary-suspension-vaccination-astra-zeneca.html.

12 Ana Catarina Pinho, *COVID-19 Vaccine AstraZeneca: Benefits Still Outweigh the Risks despite Possible Link to Rare Blood Clots with Low Platelets* (Text. European Medicines Agency) https://www.ema.europa.eu/en/news/covid-19-vaccine-astrazeneca-benefits-still-outweigh-risks-despite-possible-link-rare-blood-clots (accessed Mar. 18, 2021).

13 Bevan Shields, *EU’s Drug Regulator Backs AstraZeneca Vaccine after Safety Investigation,* The Age, https://www.theage.com.au/world/europe/eu-s-drug-regulator-backs-astrazeneca-vaccine-after-safety-investigation-20210319-p57c43.html (accessed Mar. 18, 2021).

14 France 24, *EU Says “no Evidence” to Restrict AstraZeneca Jab Use*, https://www.france24.com/en/europe/20210331-eu-says-no-evidence-to-restrict-astrazeneca-jab-use (accessed Mar. 31, 2021).

15 Ana Catarina Pinho, *AstraZeneca’s COVID-19 Vaccine: EMA Finds Possible Link to Very Rare Cases of Unusual Blood Clots with Low Platelets* (Text. European Medicines Agency) https://www.ema.europa.eu/en/news/astrazenecas-covid-19-vaccine-ema-finds-possible-link-very-rare-cases-unusual-blood-clots-low-blood (accessed Apr. 6, 2021).

16 GOV.UK, *Coronavirus Vaccine - Weekly Summary of Yellow Card Reporting*  https://www.gov.uk/government/publications/coronavirus-covid-19-vaccine-adverse-reactions/coronavirus-vaccine-summary-of-yellow-card-reporting (accessed May 21, 2021); Anna Gross & Clive Cookson, *Rare Blood Clots from AstraZeneca Vaccine Mostly Affect Younger People*  https://www.ft.com/content/dfaff95b-8f64-4963-9483-494498c55863 (accessed Apr. 29, 2021).

17 Estela Miranda Francisco, *AstraZeneca’s COVID-19 Vaccine: Benefits and Risks in Context*, (Text. European Medicines Agency), https://www.ema.europa.eu/en/news/astrazenecas-covid-19-vaccine-benefits-risks-context (accessed Apr. 23, 2021).

18 Marie Scully, Deepak Singh, & Robert Lown, *Pathologic Antibodies to Platelet Factor 4 after ChAdOx1 NCoV-19 Vaccination*, 0 N. Engl. J. Med. null (Apr. 16, 2021), https://doi.org/10.1056/NEJMoa2105385; Douglas B. Cines & James B. Bussel, *SARS-CoV-2 Vaccine–Induced Immune Thrombotic Thrombocytopenia*, 0 N. Engl. J. Med., null (Apr. 16, 2021), https://doi.org/10.1056/NEJMe2106315.

19 Anton Pottegård, Lars Christian Lund, & Øystein Karlstad, et al. *Arterial Events, Venous Thromboembolism, Thrombocytopenia, and Bleeding after Vaccination with Oxford-AstraZeneca ChAdOx1-S in Denmark and Norway: Population Based Cohort Study*, 373 BMJ, 1114 (May 5, 2021), https://doi.org/10.1136/bmj.n1114.

20 BreakingNews.ie, *Explained: Where Is AstraZeneca Being Used and Where Is It Suspended*  https://www.breakingnews.ie/covid-vaccine/factbox-some-countries-limit-astrazeneca-vaccine-use-amid-concern-over-blood-clots-1107158.html (Accessed 20 December 2021).

21
*Which Countries Have Stopped Using AstraZeneca’s COVID Vaccine?*  https://www.aljazeera.com/news/2021/3/15/which-countries-have-halted-use-of-astrazenecas-covid-vaccine (accessed Apr. 1, 2021).

22 The Guardian, *Spain, Belgium and Italy Restrict AstraZeneca Covid Vaccine to Older People*, http://www.theguardian.com/society/2021/apr/08/spain-belgium-and-italy-restrict-astrazeneca-covid-vaccine-to-older-people (accessed Apr. 8, 2021).

23
*Supra* note 2.

24
*Supra* note 4

25
*Supra* note 3.

26 nationalpost, “*Messing around Is Going to Cost Lives”: AstraZeneca Developer Slams Canada’s Approach to the Vaccine’*, https://nationalpost.com/news/messing-around-is-going-to-cost-lives-astrazeneca-developer-slams-canadas-approach-to-the-vaccine (accessed May 19, 2021).

27 Science & Environmental Health Network (SEHN), *Wingspread Statement on the Precautionary Principle* (1998). For further discussion of different formulations of the precautionary principle in a pandemic context, see *infra* note 38.

28 Maheshi N. Ramasamy, Angela M. Minassian, & Katie J. Ewer, et al. *Safety and Immunogenicity of ChAdOx1 NCoV-19 Vaccine Administered in a Prime-Boost Regimen in Young and Old Adults (COV002): A Single-Blind, Randomised, Controlled, Phase 2/3 Trial*. 396 The Lancet 1979–93 (Dec. 19, 2020), https://doi.org/10.1016/S0140-6736(20)32466-1.

29 Sanjay Mishra, *Oxford-AstraZeneca Vaccine Is Cheaper than Pfizer’s and Moderna’s and Doesn’t Require Supercold Temperature*, The Conversation, http://theconversation.com/oxford-astrazeneca-vaccine-is-cheaper-than-pfizers-and-modernas-and-doesnt-require-supercold-temperature-150697 (Nov. 2020).

30 EuroNews, *Australia Buys 20 Million More Pfizer/BioNTech Vaccines for COVID-19* (Euronews) https://www.euronews.com/2021/04/09/covid-19-australia-buys-20-million-pfizer-vaccines-after-sharp-turn-from-relying-on-astraz (accessed Apr. 9, 2021).

31
*Vaccine Suspension “necessary” for Confidence – Butler* (Mar. 14, 2021) https://www.rte.ie/news/coronavirus/2021/0314/1203917-covid-vaccine/.

32 Seema K. Shah, David Wendler, & Marion Danis, *Examining the Ethics of Clinical Use of Unproven Interventions Outside of Clinical Trials During the Ebola Epidemic*, 15 Am. J. Bioethics 11–16 (Apr. 3, 2015), https://doi.org/10.1080/15265161.2015.1010996.

33 Kat Lay Yeomans, Health Editor | Adam Sage | Tom Kington | Oliver Moody | Emma. *UK Patients Cancel AstraZeneca Covid Jabs as Europe’s Mixed Messages Sow Confusion*, (sec. news) https://www.thetimes.co.uk/article/uk-patients-cancel-astrazeneca-covid-jabs-as-europes-mixed-messages-sow-confusion-6wnp0cdlw (accessed Mar. 18, 2021); Pulse Today, *GPs Dealing with Fallout from Suspension of AstraZeneca Vaccine in Europe*, https://www.pulsetoday.co.uk/news/clinical-areas/cardiovascular/gps-dealing-with-fallout-from-suspension-of-astrazeneca-vaccine-in-europe/, (Mar. 17, 2021).

34 The Guardian, *Fear That Johnson & Johnson Pause Could Heighten Vaccine Hesitancy in US*, http://www.theguardian.com/society/2021/apr/19/johnson-johnson-pause-vaccine-hesitancy-us (Apr. 19, 2021).

35
*Flu Vaccinations Down as “wobble” in Vaccine Confidence Concerns Healthcare Workers* (May 4, 2021) https://www.abc.net.au/news/2021-05-05/flu-vaccinations-down/100114142.

36 Talha Burki, *Vaccine Misinformation and Social Media*, 1 Lancet Digital Health e258–59, https://doi.org/10.1016/S2589-7500(19)30136-0 (Oct. 1, 2019).

37 Godlee, Fiona, Jane Smith, & Harvey Marcovitch. *Wakefield’s Article Linking MMR Vaccine and Autism Was Fraudulent* 342 BMJ c7452 (Jan. 6, 2011), https://doi.org/10.1136/bmj.c7452.

38 Sarah J. L Edwards, *Ethics of Clinical Science in a Public Health Emergency: Drug Discovery at the Bedside*, 13 Am. J. Bioethics 3–14 (2013), https://doi.org/10.1080/15265161.2013.813597.

39 Megan O’Driscoll, Gabriel Ribeiro Dos Santos, & Lin Wang et al. *Age-Specific Mortality and Immunity Patterns of SARS-CoV-2*, Nature, 1–6 (Nov. 2, 2020), https://doi.org/10.1038/s41586-020-2918-0.

40
*Deaths Registered Weekly in England and Wales, Provisional — Office for National Statistics* (accessed Jan. 29, 2021), https://www.ons.gov.uk/peoplepopulationandcommunity/birthsdeathsandmarriages/deaths/bulletins/deathsregisteredweeklyinenglandandwalesprovisional/weekending15january2021#deaths-registered-by-age-group).

41 Akshay Sharma, Neel S. Bhatt, & Andrew St Martin, et al, *Clinical Characteristics and Outcomes of COVID-19 in Haematopoietic Stem-Cell Transplantation Recipients: An Observational Cohort Study*, Lancet Haematol. 8 e185–93 (Mar. 1, 2021), https://doi.org/10.1016/S2352-3026(20)30429-4.

42 Office for National Statistics, *Coronavirus (COVID-19) Infection Survey, UK* (Mar. 12, 2021) https://www.ons.gov.uk/peoplepopulationandcommunity/healthandsocialcare/conditionsanddiseases/bulletins/coronaviruscovid19infectionsurveypilot/12march2021.

43 European Centre for Disease Control, *Covid-19 Surveillance Report - Week 11, 2021* (Mar. 25, 2021) file:///Users/Jonny/Downloads/surveillance_report_20210331.html.

44
*AstraZeneca: France Says under 55s to Receive Different Second Dose of the COVID-19 Vaccine | Euronews*, https://www.euronews.com/2021/04/09/astrazeneca-france-says-under-55s-to-receive-different-second-dose-of-the-covid-19-vaccine (accessed Apr. 26, 2021).

45
*Coronavirus Disease (COVID-19): Vaccines*, https://www.who.int/news-room/q-a-detail/coronavirus-disease-(covid-19)-vaccines (accessed Apr. 26, 2021).

46 Jonathan Kimmelman, *The Risk-Escalation Model: A Principled Design Strategy for Early-Phase Trials*, 24 Kennedy Inst. Ethics J. 121–39 (2014), https://doi.org/10.1353/ken.2014.0017.

47
*Id*.

48 Paul Hunter & Lorna Fewtrell, *Acceptable Risk*, in *Water Quality: Guidelines, Standards and Health* (Lorna Fewtrell and Jamie Bartram, eds., World Health Organization, 2001), https://www.who.int/water_sanitation_health/dwq/iwachap10.pdf.

49 Jonathan Kimmelman, *Valuing Risk: The Ethical Review of Clinical Trial Safety*, 14 Kennedy Inst. Ethics J. 369–93 (2004), https://doi.org/10.1353/ken.2004.0041.

50 Karin Hedenmalm & Eva Samuelsson, *Fatal Venous Thromboembolism Associated with Different Combined Oral Contraceptives*, 28 Drug Safety, 907–16 (Oct. 1, 2005), https://doi.org/10.2165/00002018-200528100-00007.

51
*Supra* note 49.

52
*AstraZeneca Vaccine: How Do You Weigh up the Risks and Benefits?* (BBC News), sec. Explainers. https://www.bbc.com/news/explainers-56665396 (Apr. 7, 2021).

53
*COVID-19 Vaccine Linked to a Reduction in Transmission — News — Public Health Scotland*, https://www.publichealthscotland.scot/news/2021/march/covid-19-vaccine-linked-to-a-reduction-in-transmission/ (accessed Apr. 26, 2021); Office for National Statistics, *Coronavirus (COVID-19) Infection Survey Technical Article: Impact of Vaccination on Testing Positive in the UK* 10 (2021) https://www.ons.gov.uk/peoplepopulationandcommunity/healthandsocialcare/conditionsanddiseases/articles/coronaviruscovid19infectionsurveytechnicalarticleimpactofvaccinationontestingpositiveintheuk/latest; Anoop V. Shah, Ciara Gribben, & Jennifer Bishop, et al. *Effect of Vaccination on Transmission of COVID-19: An Observational Study in Healthcare Workers and Their Household* (2021), https://doi.org/10.1101/2021.03.11.21253275.

54 Of course, some may argue that paternalism can be justified in this sort of context. See *infra* note 58.

55 Public Health England, *COVID-19 Vaccines: 11,700 Deaths Prevented* (May 14, 2021), https://www.gov.uk/government/news/covid-19-vaccines-11-700-deaths-prevented.

56 Julian Savulescu, Ingmar Persson, & Dominic Wilkinson, *Utilitarianism and the Pandemic*, 34 Bioethics 620–32 (2020), https://doi.org/10.1111/bioe.12771.

57 Heidi Malm, & Mark Christopher Navin, *Pox Parties for Grannies? Chickenpox, Exogenous Boosting, and Harmful Injustices*, 20 Am. J. Bioethics 45–57 (2020), https://doi.org/10.1080/15265161.2020.1795528.

58 Lynn A. Jansen & Steven Wall. *Reconsidering Paternalism in Clinical Research*, 32 Bioethics, 50–58 (Jan. 2018), https://doi.org/10.1111/bioe.12382.

59 Malm and Navin, ‘Pox Parties for Grannies?’ To be clear, Malm and Navin are discussing a policy of refraining from providing a childhood vaccination in order to benefit adults when they invoke this argument. However, it seems plausible to claim that some critics of the utilitarian approach for vaccine policy in adults that we are discussing here could raise a similar objection.

60
*Supra* note 58; David B. Resnik, *Limits on Risks for Healthy Volunteers in Biomedical Research*, 33 Theor. Med. Bioethics 137–49 (2012), https://doi.org/10.1007/s11017-011-9201-1; Annette Rid & David Wendler, *A Framework for Risk-Benefit Evaluations in Biomedical Research*, 21 Kennedy Inst. Ethics J. 141–79 (2011), https://doi.org/10.1353/ken.2011.0007; Erin T. Paquette, & Seema K. Shah, *Towards Identifying an Upper Limit of Risk: A Persistent Area of Controversy in Research Ethics*, 63 Perspect. Biol. Med. 327–45 (2020), https://doi.org/10.1353/pbm.2020.0022; *infra* note 65. For discussion of concerns about the second premise in the research context, see *supra* note 8

61
*Supra* note 58.

62
*Supra* note 8.

63
*Supra* note 58.

64
*Id*.

65 Alex John London, *Reasonable Risks in Clinical Research: A Critique and a Proposal for the Integrative Approach*, 25 Stat. Med. 2869–85 (2006), https://doi.org/10.1002/sim.2634.

